# Deep inspiration breath hold versus free breathing technique in mediastinal radiotherapy for lymphoma

**DOI:** 10.1259/bjro.20200067

**Published:** 2021-02-04

**Authors:** Orla Anne Houlihan, Guhan Rangaswamy, Mary Dunne, Christine Rohan, Louise O'Neill, Shelton Chalke, Patricia Daly, Charles Gillham, Orla McArdle

**Affiliations:** 1 St. Luke’s Radiation Oncology Network, Dublin, Ireland

## Abstract

**Objective:**

Radiotherapy plays an important role in the management of lymphoma and many patients with lymphoma are cured with treatment. Risk of secondary malignancy and long-term cardiac and pulmonary toxicity from mediastinal radiotherapy exists. Delivery of radiotherapy using a deep inspiration breath-hold (DIBH) technique increases lung volume and has the potential to reduce dose to heart and lungs. We undertook a prospective study to assess the dosimetric differences in DIBH and free breathing (FB) plans in patients requiring mediastinal radiotherapy in clinical practice.

**Methods:**

We performed both FB and DIBH planning scans on 35 consecutive patients with mediastinal lymphoma needing radiotherapy. Contours and plans were generated for both data sets and dosimetric data were compared. All patients were planned using volumetric modulated arc therapy (VMAT). Data were compared for FB and DIBH plans with each patient acting as their own control using the related-samples Wilcoxon signed rank test.

**Results:**

DIBH significantly reduced lung doses (mean 10.6 *vs* 11.4Gy, *p* < 0.0005; V20 16.8 *vs* 18.3%, *p* = 0.001) and spinal cord maximum dose (20.6 *vs* 22.8Gy, *p* = 0.001). DIBH increased breast V4 (38.5% *vs* 31.8%, *p* = 0.006) and mean right breast dose (4.2 *vs* 3.6Gy, *p* = 0.010). There was no significant difference in heart doses when the entire study cohort was considered, however, mean heart dose tended to be lower with DIBH for upper mediastinal (UM) tumours (4.3 *vs* 4.9Gy, *p* = 0.05).

**Conclusion:**

Our study describes the potential benefit of DIBH in a population reflective of clinical practice. DIBH can decrease radiation dose to lungs, heart and spinal cord, however, may increase dose to breasts. DIBH is not always superior to FB, and the clinical significance of differences in dose to organs at risk in addition to the time required to treat patients with DIBH must be considered when deciding the most appropriate radiotherapy technique for each patient.

**Advances in knowledge:**

To our knowledge, this is the largest study comparing DIBH and FB planning for patients with lymphoma receiving mediastinal radiotherapy in clinical practice. It demonstrates the impact of an increasingly common radiotherapy technique on dose to organs at risk and the subsequent potential for long-term radiotherapy side-effects.

## Introduction

Radical treatment for lymphoma, often involving a combination of chemotherapy and radiotherapy, is associated with high cure rates and favourable overall survival.*^1,2^* Mediastinal radiotherapy, however, is associated with increased risks of late effects, including pneumonitis, pulmonary fibrosis, cardiovascular disease and secondary malignancy including breast and lung cancer, which can result in morbidity and mortality for affected patients.^3–7^ Involved site and involved node radiotherapy treatments allow reduction in target volumes and utilise advanced planning techniques to minimise dose to organs at risk (OARs) and consequent late effects.^8,9^

Deep inspiration breath-hold (DIBH) is a radiotherapy technique which has been developed to take advantage of the increase in lung volume and change in heart position, usually moved away from the chest wall, during inspiration.^4^ Delivery of radiotherapy using DIBH technique has the potential to reduce dose to heart and lungs^5^ and has been explored as a means of reducing late effects for lymphoma survivors treated with mediastinal radiotherapy. Three planning studies, recruiting a combined total of 70 selected patients, have demonstrated a benefit to DIBH in this setting.^5–7^

Based on these data, we have implemented DIBH volumetric modulated arc therapy (VMAT) in our department. We undertook a prospective study to assess the dosimetric differences seen in clinical practice between DIBH and free breathing (FB) radiotherapy plans in all patients with lymphoma requiring mediastinal radiotherapy in our institution (St Luke's Radiation Oncology Network). By including all patients with a diagnosis of mediastinal lymphoma planned for radiotherapy in our institution during the study time period, we aimed to evaluate the effect of DIBH on a spectrum of volumes and disease distributions typical of clinical practice.

## Methods and materials

### Inclusion criteria

We included all patients attending our department who required mediastinal radiotherapy for curative treatment of lymphoma. All patients had a PETCT scan performed prior to commencing chemotherapy/radiotherapy treatment. At least one PETCT scan was done during or after chemotherapy prior to radiotherapy to assess response to systemic therapy. During the study period from January 2017 to September 2019, all 35 patients who met the above criteria were included.

### CT simulation

Both DIBH and FB scans were acquired on the same day for each patient. Prior to CT simulation an information session was provided by a radiation therapist during which the DIBH process was explained and educational material in the form of booklets and video demonstrations was distributed. Following coaching, the patient was invited to demonstrate two to three practice sessions of holding their breath for 15–20 s prior to entering the CT simulation room. During scanning, patients were positioned with arms by their sides and immobilised with the aid of a thermoplastic mask. The DIBH scan without contrast was acquired first. Once in position on the CT couch, the patient was coached again before the simulation scan took place. The Varian Real Time Position Management Device (RPM) was used to obtain a breathing trace for each patient and to gate each patient’s breath-hold. A tablet computer displaying the breathing trace was used as a visual aid to assist the patient in maintaining their breath hold. This was held in place within view of the patient using a flexible in-house device, adjusted to avoid collision with the head of the gantry during treatment.

45 minutes following the DIBH CT simulation scan, a FB scan with intravenous contrast was obtained. A 45 min gap between the DIBH and FB scans was used to allow sufficient time for the patient’s breathing to return to a normal cycle prior to the FB scan.

### Contouring

The clinical target volume (CTV) for each patient was delineated based on guidelines for involved site radiotherapy.^8,9^ At the time of contouring, the pre-chemotherapy and interim or post-chemotherapy PETCT scans were displayed on a monitor adjacent to the planning scan for all patients. We do not have access to DIBH PETCT scanning facilities. CTV was contoured on the FB scan with contrast initially and then duplicated on the DIBH scan (without contrast). Careful comparison side-by side and slice-by-slice was done for each CTV. Planning target volume (PTV) margins were at the discretion of the treating clinician taking individual disease distribution into consideration, but were the same for both the DIBH and FB plan for each patient. A CTV to PTV margin of 7 mm or 8 mm for mediastinal disease was used in 83% of cases. Other PTV margins used: 10 mm in all dimensions (*n* = 2), 15 mm craniocaudal and 7 mm axial dimensions (*n* = 2), 10 mm craniocaudal and 7 mm axial dimensions (*n* = 1) and 5 mm in all dimensions (*n* = 1).

We postulated that upper mediastinal (UM) tumours may benefit more from DIBH due to the inferior displacement of both heart and lungs during deep inspiration. Each patient’s CTV was classified as whole mediastinum (WM, CTV which extended ≥3 cm below the carina) or UM (CTV <3 cm below the carina). This criteria was chosen to facilitate comparison with published studies.^5,7^

Lungs, heart, thyroid gland, spinal cord and female breasts were contoured on both FB and DIBH scans for each patient. The entire heart, including pericardium, was contoured according to the atlas published by Feng et al^10^ from just inferior to the left pulmonary artery superiorly to where the heart blends with the diaphragm inferiorly. Lung contours were generated by automatic contouring in the Eclipse treatment planning system and manually edited to include only visible lung tissue. The spinal cord and thyroid gland were contoured manually. Left and right breasts were contoured with reference to the ESTRO consensus guidelines,^11^ then these contours were summed to generate a combined breast contour. We identified all CT slices containing both PTV and breast volumes and measured the craniocaudal extent of this overlap.

### Planning and dosimetric data collection

A FB and DIBH plan was created for each patient in the Eclipse planning system, Varian Medical Systems. Treatment plans were generated using a generic starting point of full arc VMAT with two arcs. Each plan was then individualised based on the PTV volume, location and size. Partial arc, two arc and three arc plans were optimised to achieve maximal coverage and conformality while minimizing the dose to OARs according to the “ALARA” principle. In our institution, we aim to keep the mean lung dose less than 20 Gy, the volume of lung receiving ≥20 Gy (V20) less than 30% and mean heart dose less than 20 Gy. These parameters are used as a guide only and occasionally the decision is made by the treating clinician to exceed these in order to achieve acceptable target coverage which would otherwise not be possible. We do not have official dose constraints for breast tissue, however, clinicians and planners are advised to aim for as low a dose as possible, as is the case for all OARs. Final beam geometry was patient dependent. Unilateral PTVs tended to be treated with half arcs while plans to treat bilateral PTVs tended to consist of full arcs. 7 cases required 3 full arcs, 7 required between 2 and 6 partial arcs and 22 were treated with 2 full arcs. Both plans were reviewed by the treating physician, who chose the preferred plan based on clinical history, dosimetry and tolerability of DIBH set up. Once the treating clinician approved the plan, the physics department completed patient-specific quality assurance on the clinical plan.

Patient demographics and disease characteristics were extracted from each patient’s record. PTV coverage, defined as percentage of PTV receiving at least 90% (V90%) and 95% (V95%) of the prescription dose, was recorded. The percentage of PTV receiving ≥107% (V107%) of the prescription dose was also recorded. Other data collected included volumes of PTV, combined lungs and heart, mean dose to lungs, heart, breasts and thyroid gland, maximum dose to spinal cord, as well as the volume of lungs and heart receiving ≥5 Gy (V5),≥10 Gy (V10),≥15 Gy (V15),≥20 Gy (V20),≥25 Gy (V25) and ≥30 Gy (V30). The volume of each individual breast receiving ≥4 Gy (V4) and combined breasts receiving ≥4 Gy (V4) and ≥10 Gy (V10) were also recorded. We selected these dosimetric parameters for OARs as they have been included in several published studies of mediastinal radiotherapy.^5–7,12^

### Statistical analysis

Data were compared for FB and DIBH plans with each patient acting as their own control using the related-samples Wilcoxon signed rank test.

### Treatment delivery

Treatment verification was done using cone beam CT scans on days 1–3 with an average move applied on day 4 and a verification scan taken on day 4. Thereafter, weekly scans were done assuming set-up was within tolerance. The DIBH treatment set-up was well tolerated overall although some patients found the combination of breath-hold and immobilisation challenging. All patients completed planned radiotherapy treatment.

## Results

Table 1 summarises the demographic characteristics for the patient cohort. Table 2 summarises the data parameters extracted.

**Table 1.** Patient demographics

		Years median (range)
	Age	28.3 (18.9–69.2)
		**Number of patients**
Gender	Male	20
	Female	15
Histological diagnosis	Hodgkin lymphoma	28
	Diffuse large B cell lymphoma	6
	Marginal zone lymphoma	1
Stage	IA	4
	IIA	17
	IIB	13
	IIIB	1
Extra mediastinal involvement	Axilla Lower neck (to larynx) Higher neck	14 12 22
Radiotherapy dose/Fractionation	24 Gy / 12 Fr	1
	30 Gy / 15 Fr	23
	36 Gy / 18 Fr	2
	40 Gy / 20 Fr	4
	40 Gy / 20 Fr (two phase)	4
	50 Gy / 25 Fr (two phase)	1
Whole *vs* Upper mediastinum	WM	26
	UM	9
Chosen radiotherapy technique	DIBH	23
	FB	12

DIBH, deep inspiration breath-hold; FB, free breathing; UM, upper mediastinal; WM, whole mediastinum.

**Table 2.** Comparison of DIBH and FB plans

		DIBH median (range)	FB median (range)	*p* value
PTV (*n* = 35)	Volume (cc)	926.9 (129.9–3320.6)	914.2 (115.8–3135.8)	0.242
	V90% (%)	100 (100–100)	100 (98.6–100)	0.180
	V95% (%)	100 (98.6–100)	100 (97.7–100)	0.798
	V107% (%)	0 (0–0)	0 (0–0)	NA
Lungs (*n* = 35)	Volume (cc)	4547.4 (2593–6498.5)	2697.1 (1473.1–4919.5)	<0.0005
	Mean (Gy)	10.6 (5.9–17.8)	11.4 (6.4–16.9)	<0.0005
	V30 (%)	1.3 (0–13.9)	1.7 (0–14.5)	0.005
	V25 (%)	9.1 (0–22.6)	11 (0–22.6)	0.001
	V20 (%)	16.8 (4.6–39.5)	18.3 (5.9–42.2)	0.001
	V15 (%)	27.2 (9.1–52.7)	30.3 (11.9–61.4)	0.001
	V10 (%)	39.7 (21.5–76.6)	44.2 (25.3–80.6)	<0.0005
	V5 (%)	60.3 (40.2–99.2)	64.6 (43.5–94.2)	<0.0005
Heart (*n* = 35)	Volume (cc)	646.4 (481.2–1010.7)	728.3 (520.3–1263.5)	<0.0005
	Mean (Gy)	9.2 (1.4–23.8)	9.8 (2–23)	0.583
	V30 (%)	4.7 (0–27.8)	4.5 (0–28.4)	0.805
	V25 (%)	14.1 (0–56.1)	11.4 (0–53.1)	0.719
	V20 (%)	18.5 (0–69.1)	17.7 (0.8–65.3)	0.652
	V15 (%)	23.9 (0–87.8)	24 (2.1–77.7)	0.725
	V10 (%)	32.3 (0–98.6)	32.8 (4.6–94.7)	0.857
	V5 (%)	46.5 (0.3–100)	49.1 (11.6–100)	0.218
Breasts (*n* = 15)	Mean (Gy)	4.4 (0.9–11.5)	4.6 (1.2–12.2)	0.187
	V10 (%)	12.9 (0.6–68.1)	14 (0.9–65)	0.379
	V4 (%)	38.5 (4.8–99.8)	31.8 (10.2–91.1)	0.006
Left breast (*n* = 15)	Mean (Gy)	4.2 (1–12.5)	4.2 (1.4–13.7)	0.752
	V4 (%)	34.7 (6–99.6)	29.3 (12.7–91.9)	0.017
Right breast (*n* = 15)	Mean (Gy)	4.2 (0.8–11.7)	3.6 (1–10.6)	0.010
	V4 (%)	37.2 (3.7–99.9)	26 (7.8–90.3)	0.005
Thyroid (*n* = 35)	Mean (Gy)	23.8 (0.3–40.1)	22.3 (0.3–43.9)	0.326
Spinal cord (*n* = 35)	Max (Gy)	20.6 (10.1–28)	22.8 (11–29.6)	0.001

DIBH, deep inspiration breath-hold; FB, free breathing; PTV, planning target volume.

### PTV

Five patients were treated with two phases and the volumes for the first phase were used in the DIBH *vs* FB comparisons. Median PTV volume was similar for both DIBH and FB plans. PTV coverage was the same for both planning techniques.

### Lung

**Figure 1.**
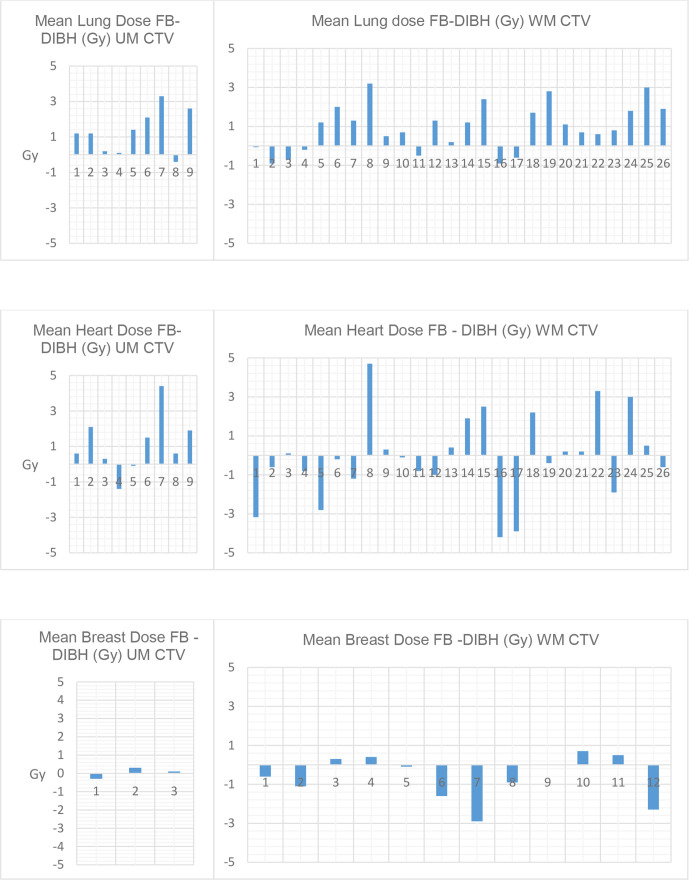
Mean DIBH doses subtracted from mean FB doses for lungs, heart and combined breasts for each individual patient. CTV, clinical target volume; DIBH, deep inspiration breath-hold; FB, free breathing; UM, upper mediastinal; WM, whole mediastinum.

DIBH was associated with an average increase of 68.6% in median lung volume (*p* < 0.0005). DIBH statistically significantly reduced the median of all measured lung parameters compared with FB. Mean lung dose was up to 29.5% higher on FB plans. There was significant variability in absolute differences in mean lung dose (Gy) as illustrated in Figure 1, but 77% of participants had reduced mean lung dose on the DIBH plan. 16 individuals had a reduction of ≥1 Gy in mean lung dose on DIBH plan.

### Heart

Median heart volume was decreased by 11.2% with DIBH versus FB (*p* < 0.0005). There were no statistically significant differences in heart doses with DIBH compared with FB. Seven of the nine individuals with UM CTV volumes had improved mean heart doses on DIBH plans as illustrated in Figure 1. Differences in mean heart dose were very variable in those with WM CTV volumes.

### Breasts

For the 15 female cases, the estimated combined mean breast dose and breast V10 were not significantly different between DIBH and FB (*p* = 0.187 and *p* = 0.379 respectively). The median mean dose was significantly larger with the DIBH plans for the right breast (*p* = 0.010). The V4 was significantly larger with the DIBH plans for both the right and left breasts (*p* = 0.005 and *p* = 0.017 respectively). Breast tissue was present on axial CT slices containing PTV for a median longitudinal length of 7 cm (range 2–10.5 cm) for DIBH plans and 5.75 cm (range 2–10 cm) for FB plans.

### Thyroid

Median mean dose to the thyroid gland was similar for both DIBH and FB plans.

### Spinal cord

The maximum dose to the spinal cord was significantly smaller with DIBH compared with FB (*p* = 0.001).

## Tumour location

The results of the separate analyses comparing mean doses to the lungs, heart and breasts for WM (*n* = 26) and UM (*n* = 9) tumours are summarised in Table 3 and absolute differences for individual cases are shown in Figure 1. This was an exploratory analysis and results should be interpreted with caution given the small number of patients in some comparisons.

**Table 3.** Comparison of mean radiation dose to the lungs, heart and female breasts for WM and UM volumes

Mean Dose (Gy)	PTV distribution	DIBH median (range)	FB median (range)	Individual patient differences (FB – DIBH)/FB (median, range (%)	*p-* value
Lungs	WM (*n* = 26)	10.9 (6.1–17.8)	12.3 (6.4–16.9)	9.6 (−7.5- + 23.2)	0.001
	UM (*n* = 9)	7.6 (5.9–10.8)	8.8 (6.4–14.1)	12.8 (−5.6- + 29.5)	0.021
Heart	WM (*n* = 26)	11.9 (4.8–23.8)	11.3 (4.1–23)	−1.9 (−102.4–+44.8)	0.611
	UM (*n* = 9)	4.3 (1.4–8.7)	4.9 (2–7.7)	22.7 (−19.2 –+72.1)	0.050
Breasts	WM (*n* = 12)	5.7 (2.2–11.5)	4.8 (1.8–12.2)	−9.9 (−88.9 –+10.6)	0.110
	UM (*n* = 3)	2 (0.9–2.4)	2.1 (1.2–2.1)	4.8 (−14.3- + 25)	0.785
Left breast	WM (*n* = 12)	5.4 (2–12.5)	5.0 (1.9–13.7)	0 (−78.9- + 16.8)	0.507
	UM (*n* = 3)	2.4 (1.0–2.8)	2.6 (1.4–2.8)	14.3 (−7.7- + 28.6)	0.276
Right breast	WM (*n* = 12)	4.8 (2.3–11.7)	4.0 (1.7–10.6)	−28.2 (−100- + 19.2)	0.012
	UM (*n* = 3)	1.6 (0.8–2.1)	1.4 (1.0–1.5)	−14.3 (−40- + 20)	0.414

DIBH, deep inspiration breath-hold; FB, free breathing; PTV, planning target volume; UM, upper mediastinal; WM, whole mediastinum.

DIBH plans had statistically significantly smaller mean doses to lungs for both WM and UM tumours. DIBH was also associated with a reduced median mean heart dose for UM tumours (*p* = 0.050).

There was no significant difference in dose to combined breasts with DIBH compared with FB for WM or UM tumours. The median mean dose to the right breast was significantly greater with the DIBH plans for the WM tumours, however, absolute difference was small (0.8 Gy) (*p* = 0.012).

### Reasons for choosing FB plan

**Figure 2.**
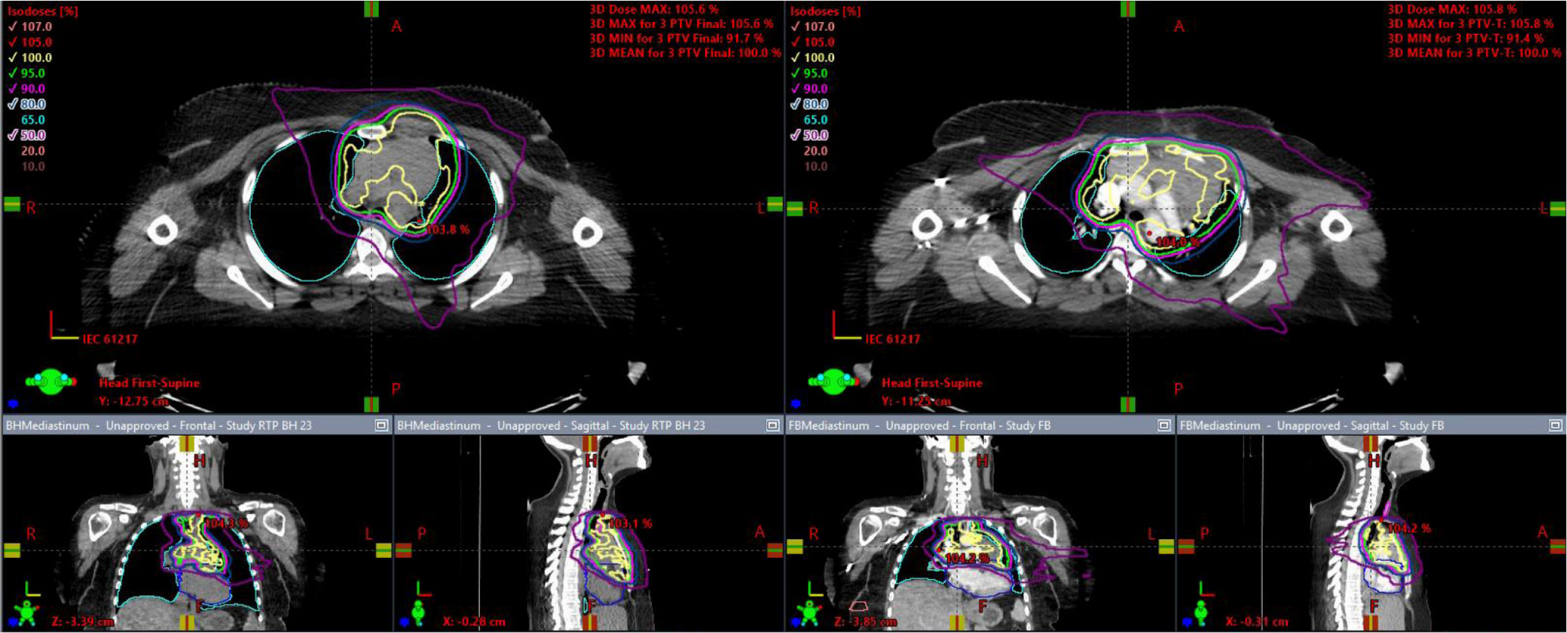
DIBH plan (left) with dosimetric advantage over FB plan (right). DIBH, deep inspiration breath-hold; FB, free breathing.

**Figure 3.**
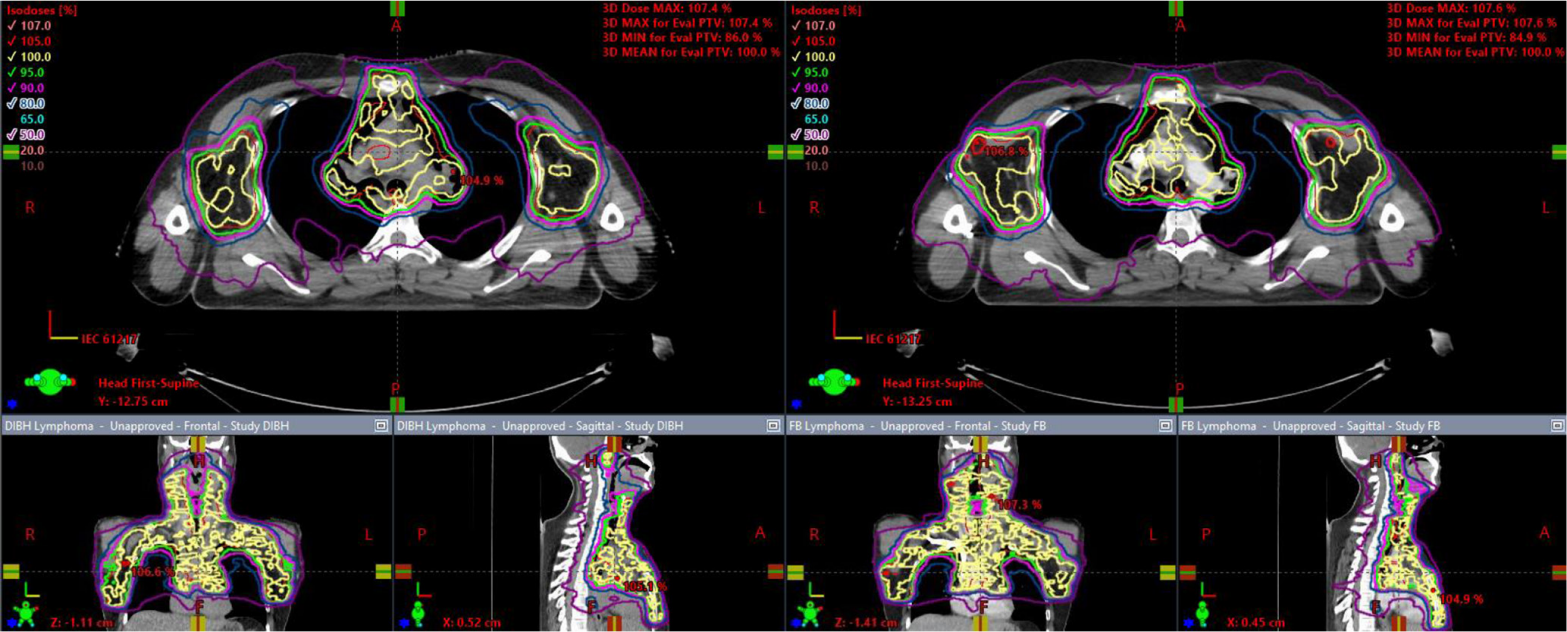
FB (right) plan with marginal dosimetric advantage over DIBH plan (left). DIBH, deep inspiration breath-hold; FB, free breathing.

12 patients were treated using the FB plan. Reasons for this were difficulty tolerating DIBH (*n* = 3), lack of a worthwhile clinical difference between the DIBH and FB plans as determined by the treating clinician (*n* = 6), and marginally improved dose to OARs with the FB plan (*n* = 3). Figure 2 illustrates an example of a plan in which DIBH was associated with a clear dosimetric advantage and Figure 3 illustrates a plan in which FB was preferred.

## Discussion

To our knowledge, this is the largest study comparing DIBH and FB planning for patients with lymphoma receiving mediastinal radiotherapy in clinical practice. In our study, DIBH was associated with significantly reduced lung and spinal cord doses compared with FB. There was no significant difference in heart dose between DIBH and FB. V4 of both individual and combined breasts was increased with DIBH. DIBH also increased mean right breast dose compared with FB. Our patient population is less highly selected than most in the published literature. Paumier et al^7^ included only patients with Stage I/II Hodgkin lymphoma and volumes were contoured according to involved node treatment guidelines resulting in PTV volumes smaller than those in our study (median 720 cc DIBH and 750 cc FB). Starke et al^6^ contoured volumes according to involved site radiotherapy as in our study but they excluded patients with high neck, axillary or extra nodal disease, reporting much smaller median PTV volumes (median 405 cc DIBH and 611 cc FB). Our study included patients with both high neck (*n* = 22) and axillary (*n* = 14) disease. Petersen et al^5^ similar to our design included patients requiring mediastinal radiotherapy without exclusions regarding involved sites. Target volumes were contoured according to involved node treatment guidelines and they report similar PTV volumes to our study (median 945 cc DIBH and 1198 cc FB).

Petersen et al^5^, Starke et al^6^, and Paumier et al^7^ have all conducted planning studies comparing DIBH to FB mediastinal radiotherapy for lymphoma. Planning techniques used by Petersen et al^5^ were either parallel opposing fields with or without supplementary fields to reduce hot spots or intensity-modulated radiotherapy (IMRT). Starke et al^6^ created both full and butterfly VMAT plans for each patient, and Paumier et al^7^ used IMRT with five equidistant coplanar beams. In these studies, DIBH was associated with a significant reduction in radiation dose to the lungs and heart, for all measured dosimetric parameters.

As in previously published data above, DIBH in our study reduced lung dose compared with FB. We found a highly statistically significant difference in median mean lung dose (0.8 Gy reduction with DIBH, *p* < 0.001). This compares with values of 1.3–2.4 Gy reductions in mean lung dose in the above-mentioned studies. The clinical significance of a reduction of 0.8 Gy in mean lung dose is arguable, however there was a wide range in absolute difference for individual cases as summarised in Table 3 and illustrated in Figure 1. Several studies have reported dose–response curves for radiation pneumonitis. While results vary, reported data suggest that the risk of pneumonitis increases with increasing mean lung dose.^13^ Given the additional risk of pneumonitis with chemotherapeutic agents such as bleomycin,^14^ it is likely that individuals in our study derived real clinical benefit from treatment with DIBH.

While the mean heart dose was lower for our DIBH plans (9.2 *vs* 9.8 Gy on FB plans), this reduction was not statistically significant. We found a highly significant 11.2% reduction in median heart volume for DIBH plans (728 cc *vs* 646 cc, *p* < 0.0005), while PTV volume was not significantly different between DIBH and FB plans. For WM DIBH plans, therefore, a similarly sized PTV extending anterior to a smaller heart volume results in no advantage for mean heart dose (median mean heart dose 5.3% higher for DIBH plans, *p* = 0.611). The only other paper to report heart volume for both plans was Starke et al^6^ who demonstrate an 8% reduction in heart volume for DIBH plans.

We postulated that UM DIBH plans would reduce mean heart dose as the heart volume, although smaller, moves away from the PTV with inspiration. There was a trend in this direction (median mean heart dose 4.3 Gy with DIBH *vs* 4.9 Gy with FB, *p* = 0.05), although the small number of patients makes it impossible to draw any definitive conclusions. In the two studies which also contained this subset analysis of UM *vs* WM tumours, results are conflicting. Petersen et al^5^ report a more pronounced advantage with DIBH for patients with WM tumours while Paumier et al^7^ report a greater benefit for UM tumours, as in our study. Of note, Petersen et al^5^ reduced the PTV margin on DIBH plans while Paumier et al,^7^ similar to our study, did not.

In our study, the CTV to PTV margin was the same for each patient’s DIBH and FB plan. Paumier et al^7^ applied the same margin (10 mm CTV to PTV) for both FB and DIBH plans, but were able to achieve significant reductions in mean heart doses for DIBH plans in a selected group of patients. Starke et al^6^ reduced PTV margins by 5 mm in all directions for DIBH plans resulting in median PTV volumes of 611 and 405 cc respectively. This reduction of 33% in PTV volume with DIBH undoubtedly contributed to the sparing of organs at risk. Our study included patients with both high neck (*n* = 22) and axillary (*n* = 14) disease. PTV volumes in our study are similar to those in the study by Petersen et al,^5^ however Petersen et al reduced PTV margins by 5 mm in the craniocaudal direction for DIBH plans, resulting in significantly smaller PTV volumes for DIBH plans (median 945 cc DIBH and 1198 cc FB). The identical margins used for both DIBH and FB plans for each patient, in addition to the larger PTV volumes included, are likely to contribute to the lack of benefit in heart dose seen with DIBH in our study, in contrast with previously published studies.^5–7^

The higher breast doses with DIBH in our study may be due to the anatomical change of breast position during inspiration. We observed an increased overlap of axial CT slices containing both breast tissue and PTV in DIBH compared with FB (7 *vs* 5.75 cm in longitudinal dimensions respectively). There was no significant difference in mean breast dose in the study by Petersen et al^5^, however, there was a non-significant increase in mean right breast dose with DIBH compared with FB (6.4 *vs* 5.0 Gy). DIBH did significantly reduced mean, V10 and V4 breast doses in full arc VMAT plans in the study by Starke et al^6^, although as previously mentioned, PTV margins for DIBH plans were smaller than in our study which may also account for our discordant results.

The three cases in our study for which FB was associated with a marginal benefit to DIBH had WM tumours, high neck disease and DIBH PTV volumes that were greater than the median for the study (1071–3320 cc). The PTV in two of the three cases also included the axilla. While it is impossible to draw any conclusions given the small number of cases, it may be the case that it is not possible to realise the full potential of DIBH treatment delivery for larger mediastinal volumes unless the PTV margin is reduced. We suggest DIBH to be of advantage when used to treat PTV volumes less than 1000cc, as was the case in the papers published by Paumier et al.,^7^ Starke et al^6^ and Petersen et al.^5^ In our initial implementation phase, we chose to deliver DIBH plans using the same PTV margin as FB plans. Given successful delivery of all DIBH treatment plans, we now intend to assess the feasibility of reducing PTV margins for these cases. Practically, the reduction in PTV margins for DIBH plans should result in a greater dosimetric advantage for this radiotherapy technique.

In our study, all plans were generated using full or partial arc VMAT. While 3D conformal radiotherapy (3DCRT) can achieve target coverage in mediastinal radiotherapy planning, the use of IMRT can result in a more conformal plan, with improved homogeneity and fewer hot spots, although this is often at the expense of increased low dose to OARs.^15^ Potential techniques to optimise tumour coverage while minimising dose to OARs include butterfly IMRT^16^ and VMAT techniques^15^ which employ 5–7 beams or multiple arcs respectively with restricted entry angles to deliver radiation through the centre of the thorax and avoid beam entry through lateral lung segments or female breasts. Compared with full arc VMAT, butterfly techniques have been shown to reduce the low dose bath of radiation to the lungs and breasts, however, increased higher radiation doses (V20, V25, V30) to the lungs have also been reported.^6,15^ In addition, as Starke et al^6^ report, the limited delivery angles can lead to reduced radiotherapy plan homogeneity, the accumulation of hotspots in the sternum and reduced cardiac-sparing. Butterfly VMAT also takes longer to deliver than full arc VMAT due to the increased number of beams and the requirement for a couch rotation. It is therefore essential that individual patient characteristics including age, sex, anatomy and tumour distribution are taken into consideration when determining the most appropriate radiotherapy technique.

All patients in our study were compliant with DIBH at CT simulation, although some found it challenging due to anxiety associated with adhering to the breath-hold technique and feeling enclosed by the thermoplastic mask. Initially we offered the same coaching session and information booklet to patients as used for DIBH in breast radiotherapy, which has been implemented in our institution for several years. In response to the challenges of patient tolerability of DIBH with mediastinal radiotherapy, we increased our coaching time, rewrote patient information booklets, provided instructional videos and offered the assistance of a psychologist to coach patients on-set, if required. These interventions were successful in the majority of cases. As previously mentioned, three patients were unable to tolerate DIBH during treatment delivery, despite these interventions, and so were treated with the FB scan. We have found it to be of utmost importance to ensure patients understand prior to commencing the planning process that DIBH is just one of a range of tools used to produce an optimal radiation treatment plan in order to avoid significant psychological morbidity if the DIBH protocol is not tolerated.

The delivery of radiotherapy using DIBH requires more time than FB. In our experience, coaching prior to CT simulation requires 20–40 min of individualised patient care and the delivery of each radiotherapy fraction is extended by 10–20 min, depending on the patient’s ability to maintain a stable breath hold. It is therefore important to ensure that adequate time is allocated to patients treated with DIBH during radiotherapy.

Our study is strengthened by the large number of patients included. It offers an insight into the practicalities of mediastinal radiotherapy planning and treatment in real clinical practice. A potential limitation of our study is that DIBH scans were obtained without contrast and FB scans were obtained with intravenous contrast. The use of intravenous contrast can improve visualisation and accuracy of delineation of radiotherapy target volumes and OARs which may have resulted in discrepancies in contours between DIBH and FB scans.^17^ Our contouring protocol, however, seeks to minimise this difference by reviewing both DIBH and FB scans side-by side and slice-by-slice. We do not have access to PETCT scans performed in DIBH to allow accurate fusion during target volume delineation.

## Conclusions

Our study describes the effect of DIBH on dose to OARs for patients with lymphoma who received mediastinal radiotherapy at our institution over a 33 month period. 23 patients (66%) were treated using DIBH, with a significant reduction in mean lung dose and spinal cord dose for the entire group. DIBH was associated with increased mean breast dose in female patients. There was significant variation in absolute benefits to mean lung and heart dose. DIBH is not always superior to FB, potentially for patients with long, complex volumes when similar PTV margins are used and where dosimetrially beneficial may only result in a small reduction in dose. The clinical significance of differences in dose to organs at risk in addition to the time required to treat patients with DIBH must be considered when deciding the most appropriate radiotherapy technique for each patient.
